# Exogenous Melatonin Enhances the Salt Tolerance of Celery (*Apium graveolens* L.) by Regulating Osmotic Adaptation and Energy Metabolism via Starch and Sucrose Metabolic Pathways

**DOI:** 10.3390/ijms27031299

**Published:** 2026-01-28

**Authors:** Zhiheng Chen, Wenhao Lin, Shengyan Yang, Wenjia Cui, Shiyi Zhang, Zexi Peng, Yonglu Li, Yangxia Zheng, Fangjie Xie, Mengyao Li

**Affiliations:** 1 Xinjiang Production & Construction Corps Key Laboratory of Protected Agriculture, Tarim University, Alar 843300, China; mwvmg9218@163.com (Z.C.); 1101223213@stumail.taru.edu.cn (S.Y.); 10757242092@stumail.taru.edu.cn (Z.P.); 2041222125@stumail.taru.edu.cn (Y.L.); 2 College of Horticulture, Sichuan Agricultural University, Chengdu 611130, China; m15397628813@163.com (W.L.); 18543518311@163.com (W.C.); 18292767970@163.com (S.Z.); zhengyx13520@sicau.edu.cn (Y.Z.); 3 College of Horticulture and Forestry, Tarim University, Alar 843300, China

**Keywords:** *Apium graveolens* L., melatonin, osmotic regulation, transcriptome analysis, carbon metabolism, salt tolerance

## Abstract

Salt stress is one of the main abiotic stresses that restrict crop production. Melatonin (MT), a signal molecule widely present in plants, plays an important role in regulating abiotic stress response. In this study, celery seedlings were used as experimental materials, and the control, salt stress, and exogenous MT treatment groups under salt stress were set up. Through phenotypic, physiological index determination, transcriptome sequencing, and expression analysis, the alleviation effects of MT on salt stress were comprehensively investigated. The results showed that exogenous MT treatment significantly reduced seedling growth inhibition caused by salt stress. Physiological measurements showed that MT significantly reduced malondialdehyde content, increased the activities of superoxide dismutase (SOD), peroxidase (POD) and catalase (CAT), promoted the accumulation of free proline and soluble protein, and increased photosynthetic parameters such as chlorophyll, ΦPSII, Fv/Fm, and ETR. Transcriptome analysis showed that MT regulates the expression of several genes associated with carbon metabolism, including β-amylase gene (*AgBAM*), sucrose-degrading enzyme genes (*AgSUS*, *AgINV*), and glucose synthesis-related genes (*AgAG*, *AgEGLC*, *AgBGLU*). The results of qRT-PCR verification were highly consistent with the transcriptome sequencing data, revealing that MT synergistically regulates starch and sucrose metabolic pathways, and effectively alleviates the damage of celery seedlings under salt stress at the molecular level. In summary, exogenous MT significantly improved the salt tolerance of celery by enhancing antioxidant capacity, maintaining photosynthetic function, promoting the accumulation of osmotic adjustment substances, and synergistically regulating carbon metabolism-related pathways. The concentration of 200 μM was identified as optimal, based on its most pronounced alleviating effects across the physiological parameters measured. This study provides an important theoretical basis for utilizing MT to enhance plant salt resistance.

## 1. Introduction

Drought caused by global warming, soil salinization caused by sea level rise, and the irrigation of high-salinity groundwater have aggravated soil salinity, significantly constraining crop growth and yield formation [1]. Salt stress disrupts the normal physiological processes of plants through multiple mechanisms [2]. Excessive soluble salt in the soil can lower water potential, inhibit root water absorption, cause osmotic stress, and destroy ion homeostasis [3]. Furthermore, salt stress can lead to stomatal closure and inhibit the activity of key enzymes involved in carbon assimilation, thereby reducing photosynthetic efficiency [4]. Additionally, salt stress can induce excessive accumulation of reactive oxygen species in cells, causing oxidative damage to macromolecules such as lipid membranes, nucleic acids, proteins, essential enzymes, carbohydrates, and photosynthetic pigments, leading to cell redox imbalance and seriously interfering with energy metabolism and material transformation [5].

To cope with salt stress, plants have developed multi-level adaptive strategies in long-term evolution. Studies have shown that salt stress can induce structural adaptive changes such as smaller and thicker leaves, as well as thickened mesophyll tissue and epidermal cell layers [6]. Different salt-tolerant plants adapt to high-salinity environments by adjusting the biomass allocation between aboveground parts and roots, while salt-sensitive plants often reduce root biomass to limit salt absorption [7]. Concurrently, plants remove excessive reactive oxygen species (ROS) and reduce oxidative damage by activating their antioxidant enzyme systems. To adapt to salt conditions, plants also maintain cellular osmotic balance by accumulating osmotic regulators such as proline, betaine, and soluble sugar [8]. In addition, under salt stress, plants regulate the transcription of numerous downstream functional genes by upregulating transcription factors such as *NAC*, *MYB*, *bHLH*, and *ERF*. This regulation participates in a series of defense responses, including signal transduction, osmotic protector synthesis, and antioxidant system construction, thereby systematically enhancing plant survival and adaptability in high-salinity environments [9].

Melatonin (N-acetyl-5-methoxytryptamine, MT), an indole heterocyclic compound prevalent in both animals and plants, has been recognized as a crucial endogenous signaling molecule [10,11,12]. Studies have shown that MT not only participates in the regulation of seed germination, fruit development, mineral nutrition absorption, and other physiological processes, but also significantly enhances the adaptability of plants to various stresses such as drought, extreme temperature, heavy metals, and salt stress [13,14,15,16]. Exogenous application of MT activates the ROS scavenging system, increases the concentration of osmotic adjustment substances, and reduces the membrane lipid peroxidation level, thereby enhancing the salt tolerance of crops such as apples [17] and corn [18]. MT can also further promote the expression of salt-tolerance genes in cucumber by regulating the expression of key genes such as *HSPs*, *MYB*, and *bZIP* [19]. In addition, MT promotes the accumulation of soluble sugar by affecting the expression of key genes involved in starch and sucrose metabolic pathways, thereby further enhancing the osmotic adjustment ability of maize [20].

Celery (*Apium graveolens* L.) is one of the most important vegetable crops in the Apiaceae family, originating from the Mediterranean coast and now extensively cultivated worldwide [21]. Celery is rich in various bioactive compounds, including apigenin, carotene, and flavonoids, which contribute to its pharmacological functions such as anti-oxidation, blood pressure reduction, lipid regulation, and potential anti-cancer effects [22]. However, the roots of celery are characterized as short and shallow, resulting in limited water absorption capacity and heightened sensitivity to salt stress. Salt stress not only inhibits its growth and photosynthesis but also damages the photosynthetic apparatus and cell membrane integrity, resulting in reduced quality [23,24]. Research has demonstrated that the application of exogenous aspartic acid and proline can enhance the salt tolerance of celery, which provides theoretical support for the stress-resistant cultivation techniques [25,26]. While exogenous MT has been shown to effectively enhance plant resistance to salt stress by activating the antioxidant system, its effects on celery have not yet been reported. The objective of this study was to systematically analyze the physiological and molecular regulatory effects of exogenous MT on celery seedlings subjected to salt stress, with the aim of elucidating the mechanism of MT improving salt tolerance in celery. The findings offer a theoretical basis for the application of MT in enhancing stress resistance in vegetable production.

## 2. Results

### 2.1. Effects of Exogenous MT on Growth Parameters of Salt-Stressed Celery Seedlings

**Figure 1:**
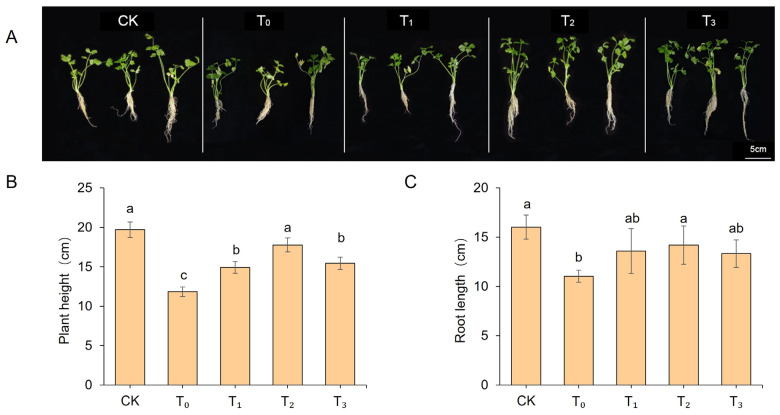
Phenotypic and growth parameters of celery seedling under different treatments. (**A**) Phenotypic characteristics of celery seedlings under salt stress. (**B**) Plant height. (**C**) Root length. Data are presented as mean ± SD (*n* = 3). Different lowercase letters above bars indicate statistically significant differences among treatments as determined by one-way ANOVA followed by Duncan’s multiple range test (*p* < 0.05). CK: Blank control, T_0_: salt stress treatment, T_1_: 100 μM exogenous MT + salt stress, T_2_: 200 μM exogenous MT + salt stress, T_3_: 300 μM exogenous MT + salt stress.

As shown in Figure 1, the control group (CK) exhibited normal growth patterns. Following a 7-day period of salt stress treatment, significant phenotypic variations were observed among the experimental groups. The salt-stressed group (T_0_, 0 μM MT) exhibited noticeable leaf wilting and yellowing, with growth severely inhibited. Conversely, the application of exogenous MT treatments alleviated salt damage symptoms to varying degrees, with the 200 μM MT treatment group (T_2_) showing the best growth status. As shown in Figure 1B,C, compared to CK, the T_0_ group exhibited significant reductions in plant height and root length, with decreases of 39.95% and 31.19%, respectively. Exogenous MT treatment mitigated these declines, with the 200 μM MT (T_2_) treatment yielding significantly superior results across all parameters compared to T_3_ and T_1_ groups. These findings indicate that exogenous MT treatment effectively alleviates salt stress-induced growth inhibition in celery seedlings, with the 200 μM concentration exhibiting the most pronounced effect in this study.

### 2.2. Effects of Exogenous MT on Physiological Parameters of Celery Leaves Under Salt Stress Treatment

**Figure 2:**
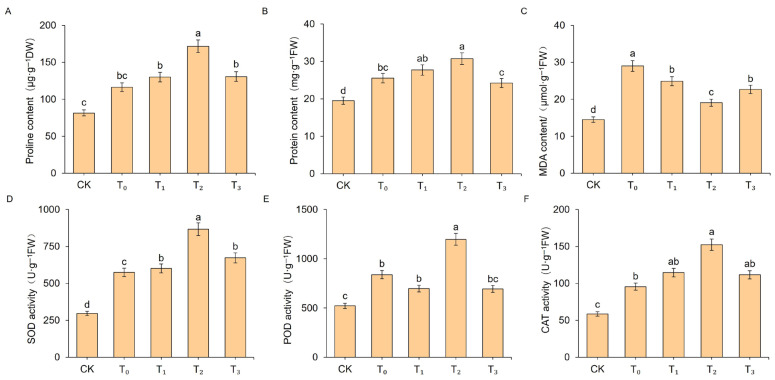
Physiological indicators of celery seedling leaves under different treatments. (**A**) Proline (Pro), (**B**) soluble protein, (**C**) malondialdehyde (MDA), (**D**) superoxide dismutase (SOD), (**E**) peroxidase (POD), and (**F**) catalase (CAT). Data are presented as mean ± SD (*n* = 3). Different lowercase letters above bars indicate statistically significant differences among treatments as determined by one-way ANOVA followed by Duncan’s multiple range test (*p* < 0.05). CK: blank control, T_0_: salt stress treatment, T_1_: 100 μM exogenous MT + salt stress, T_2_: 200 μM exogenous MT + salt stress, T_3_: 300 μM exogenous MT + salt stress.

As illustrated in Figure 2, compared with the CK group, the T_0_ group exhibited significantly elevated levels of Pro, soluble protein, and MDA in celery seedlings, with increases of 27.11%, 21.10%, and 49.99%, respectively. Exogenous MT influenced the accumulation of osmotic regulators and membrane lipid peroxidation products. Pro and soluble protein levels in all treatment groups initially increased and then decreased with rising exogenous MT concentrations, whereas MDA levels showed the opposite trend (Figure 2A–C). The most pronounced effect was observed at 200 μM, where Pro and soluble protein contents increased by 36.67% and 18.10%, respectively, compared to the T_0_ group, while MDA content decreased by 34.31%. Salt stress significantly enhanced SOD, POD, and CAT activities, and exogenous MT treatment further amplified these enzyme activities, with all reaching peak levels at 200 μM (Figure 2D–F). In comparison to the T_0_ group, SOD, POD, and CAT activities in the T_2_ group increased by 33.65%, 29.92%, and 37.27%, respectively. In summary, exogenous MT application effectively promoted the accumulation of osmotic regulatory substances, inhibited membrane lipid peroxidation, and synergistically enhanced the activity of the antioxidant enzyme system, with the most pronounced ameliorative effect observed at 200 μM MT.

### 2.3. Effects of Exogenous MT on Chlorophyll and Fluorescence Parameters in Salt-Stressed Celery

**Figure 3:**
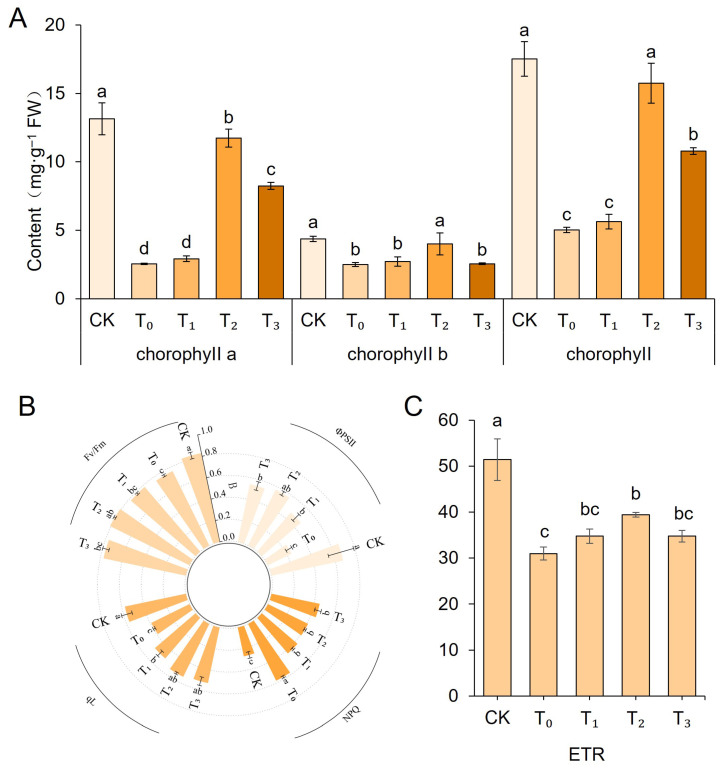
Chlorophyll and fluorescence parameters in celery seedling leaves under different treatments. (**A**) Chlorophyll content under salt stress. (**B**,**C**) Fluorescence kinetic parameters. Data are presented as mean ± SD (*n* = 3). Different lowercase letters above bars indicate statistically significant differences among treatments as determined by one-way ANOVA followed by Duncan’s multiple range test (*p* < 0.05).

As shown in Figure 3A, compared with the CK group, salt stress severely inhibited the synthesis of photosynthetic pigment in celery. The T_0_ group exhibited reductions of 80.65%, 43.10%, and 71.28% in Chla, Chlb, and total chlorophyll content, respectively. Exogenous MT effectively mitigated chlorophyll reduction, with the most pronounced effect observed at a concentration of 200 μM. Compared to the T_0_ group, the Chla, Chlb, and total chlorophyll content increased by 78.34%, 37.78%, and 68.04%, respectively. Furthermore, salt stress significantly inhibited the photochemical efficiency of PSII (Figure 3B). Compared with the CK group, the T_0_ group exhibited significant declines in Φ PSII, Fv/Fm, qL, and ETR, while NPQ was significantly elevated. Exogenous MT effectively mitigated the reductions in ΦPSII, Fv/Fm, qL, and ETR, while suppressing the increase in NPQ. These results indicate that exogenous MT can effectively improve the photosynthetic performance of celery under salt stress by maintaining higher chlorophyll content and protecting the functional integrity of PSII.

### 2.4. Transcriptome Sequencing Analysis and DEG Screening

Transcriptome sequencing was conducted to further investigate the mechanism by which exogenous MT alleviates salt stress. Three sample groups (CK, T_0_, and T_2_) were selected to represent the control (CK), salt stress (Salt), and MT + salt stress (MTSalt) treatments, respectively. Each treatment group comprised three biological replicates, resulting in a total of nine cDNA libraries for transcriptome sequencing. The sequencing yielded 64.97 G of raw data, with average Q20 and Q30 coverage exceeding 99.28% and 97.85%. Principal component analysis revealed clear sample separation and clustering, indicating high-quality sequencing suitable for subsequent analysis (Figure S1).

**Figure 4:**
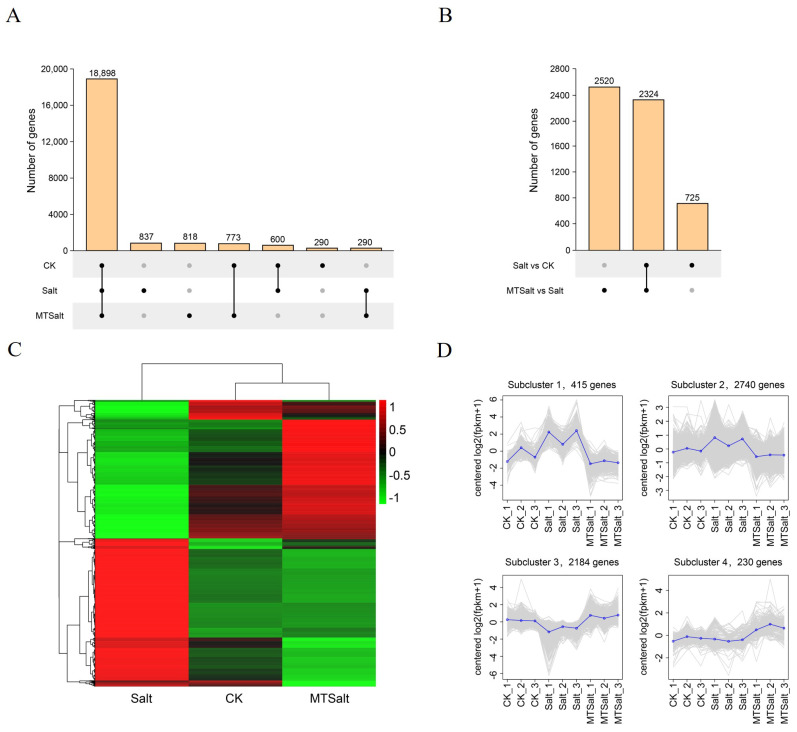
Expression and clustering analysis of differentially expressed genes. (**A**) UpSet plot analysis of gene expression counts in CK, Salt, and MTSalt groups. (**B**) UpSet plot analysis of DEGs between Salt vs. CK and MTSalt vs. Salt control pairs. (**C**) Clustering analysis of differential gene transcription abundance across the three treatment groups. (**D**) K-means clustering of gene expression trends. Expression profiles for each gene within each subcluster are depicted as gray lines, while the average expression profile across all genes in each sample is shown in blue.

DEGs were identified across treatments based on FPKM normalization and differential expression thresholds. As shown in Figure 4A, a total of 18,898 genes were co-expressed across all three treatments, reflecting the basal transcriptomic composition of celery under diverse environmental conditions. Further analysis identified genes that were both co-expressed and uniquely expressed between the Salt vs. CK and MTSalt vs. Salt control groups. The MTSalt vs. Salt comparison contained 1795 more DEGs than the Salt vs. CK comparison and included 2520 uniquely expressed genes (Table S1). This suggests these genes may be key regulators responding to salt stress under exogenous MT induction (Figure 4B and Figure S1).

Cluster analysis revealed significant differences in the expression of DEGs across various samples (Figure 4C), with a large number of DEGs detected as highly expressed in the Salt vs. MTSalt group. K-means clustering further categorized DEGs into four subclusters. Subcluster 1 (415 genes) and Subcluster 2 (2740 genes) were upregulated in the Salt group but downregulated in the MTSalt group, whereas genes in Subclusters 3 and 4 were upregulated in the MTSalt group (Figure 4D). These results indicate that salt stress significantly promotes the expression of certain genes, while exogenous MT treatment significantly reverses some salt-induced gene expression changes and specifically activates another subset of genes. This suggests that exogenous MT may regulate celery’s adaptation to salt stress by modulating the expression of specific genes.

### 2.5. Functional Annotation of DEGs

**Figure 5:**
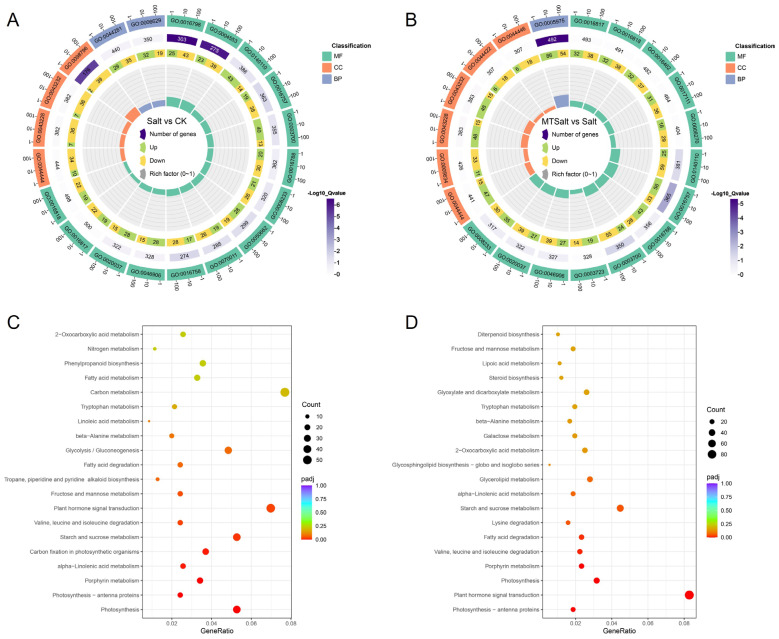
Circular diagram of GO functional annotation for DEGs and KEGG metabolic pathway analysis. (**A**) GO enrichment circle diagram for DEGs in “Salt vs. CK”. (**B**) GO enrichment circle diagram for DEGs in “MTSalt vs. CK”. (**C**) KEGG pathway analysis for DEGs in “Salt vs. CK”. (**D**) KEGG pathway analysis for DEGs in “MTSalt vs. CK”. GO Circular Diagram: Blue represents biological process, orange represents cellular component, and green represents molecular function. Purple-Log10(Q-value) indicates enrichment level, with higher values denoting more significant enrichment. The outer scale from 1 to 100 shows the number of enriched genes. KEGG Pathway Enrichment Plot: The vertical axis displays pathway annotation information, while the horizontal axis shows the corresponding Rich factor. Q-value magnitude is represented by dot color—smaller Q-values appear closer to red. Dot size indicates the number of DEGs within each pathway. For both GO and KEGG analyses, the top 20 terms with the highest enrichment were selected for plotting.

To elucidate the biological functions of DEGs, GO and KEGG analysis were performed on DEGs from the Salt vs. CK and MTSalt vs. Salt comparison groups (Table S2). As shown in Figure 5A,B, within the top 20 most enriched GO terms, the Salt vs. CK group exhibited significant enrichment in molecular function categories related to anhydrase activity such as GO:0016817 and GO:0016818, each encompassing 41 DEGs. In contrast, the MTSalt vs. Salt group showed significant enrichment in biological process categories for carbohydrate metabolism pathways (GO:0005975, 140 DEGs). KEGG pathway enrichment analysis revealed that DEGs in both comparison groups were significantly enriched in pathways including photosynthesis, carbon metabolism, starch and sucrose metabolism, and plant hormone signaling (Figure 5C,D). Among these, starch and sucrose metabolism and plant hormone signaling showed the highest enrichment levels and the largest number of DEGs, suggesting their potential role as key pathways in the mitigation of salt stress in celery mediated by MT.

### 2.6. Identification of Differentially Expressed Transcription Factor Genes

**Figure 6:**
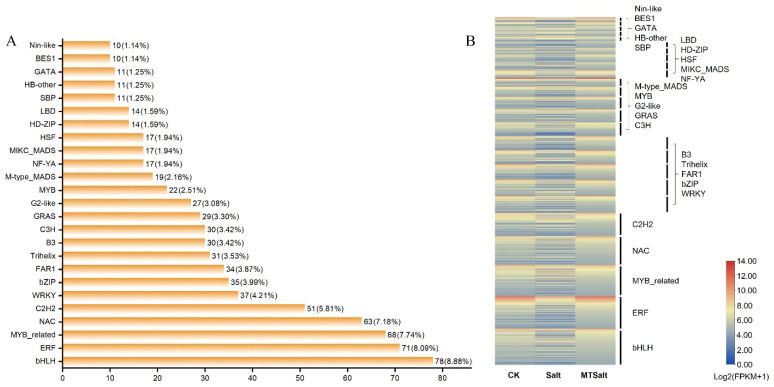
Transcription factor analysis of differentially expressed genes. (**A**) Names of transcription factor families and corresponding numbers of transcription factors. (**B**) Specific expression patterns of transcription factors, with colors ranging from blue to red indicating expression levels from low to high, calculated using log2(FPKM + 1). The figure displays the top 25 transcription factor families with higher expression levels.

This study successfully identified 3387 transcription factors from differentially expressed genes (DEGs), categorized into 57 distinct families. The number of members and their expression patterns exhibited significant specificity to each family (Figure 6A,B). Among them, the bHLH family contained the largest number of members (78, accounting for 8.88%), followed by the ERF (71, 8.09%), MYB_related (68, 7.74%), and NAC (63, 7.18%) families. Collectively, these four families constituted 32% of all identified transcription factors, forming the core transcription factor families involved in the celery salt stress response DEGs. Expression heatmaps further illustrated the expression patterns of these core transcription factor families under different treatments. Families such as bHLH, NAC, and ERF consistently exhibited high expression levels across all treatments, while showing distinct regulatory patterns in the Salt group compared to CK and MTSalt group. These findings suggest that key transcription factor families, especially ERF and NAC, may play crucial roles in the exogenous MT-mediated regulatory network in celery salt stress responses.

### 2.7. Analysis of the Sucrose and Starch Metabolism Pathways

**Figure 7:**
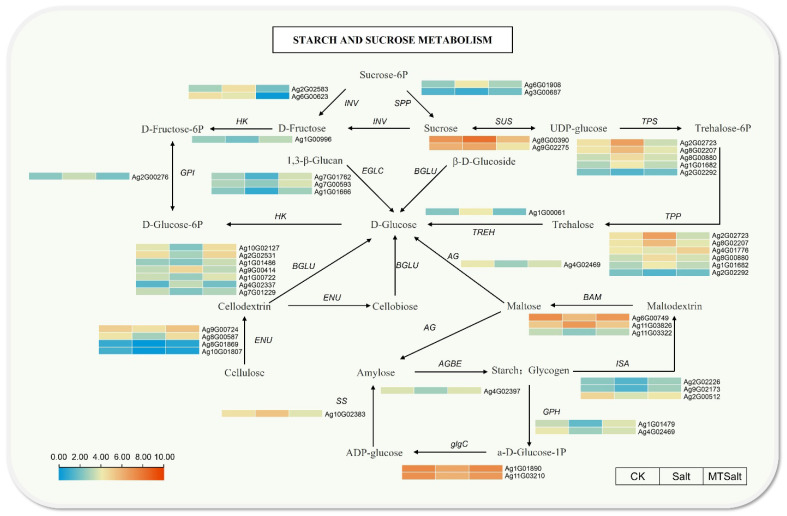
Gene expression changes in starch and sucrose metabolic pathways under salt stress. Red (upregulated) and blue (downregulated) in the heatmap indicate gene expression trends, and expression was calculated using log2(FPKM + 1).

The KEGG enrichment analysis indicates that exogenous MT treatment in celery seedlings may mitigate salt stress by regulating genes involved in starch and sucrose metabolism pathways (Table S3). Based on the starch and sucrose metabolism pathway, a pathway diagram was constructed (Figure 7), showing significant changes in the expression of most key enzyme encoding genes after MT treatment. In sucrose metabolism, the Salt group exhibited significantly increased expression of sucrose synthase genes *AgSUS* (e.g., *Ag8G00390*), and β-fructofuranosidase genes *AgINV* (e.g., *Ag2G02583*, *Ag6G00623*) showed significantly upregulated expression. Exogenous MT counteracted this trend by downregulating the expression of *AgSUS* and *AgINV*, thereby promoting sucrose accumulation. This finding suggests exogenous MT may enhance osmotic substance accumulation in celery, thereby improving stress resistance. Additionally, compared to the Salt group, exogenous MT reduced the expression of trehalose synthesis-related genes. Regarding starch metabolism, salt stress suppressed the expression of key starch synthesis enzymes, including *AgISA* and *AgAGBE*, whereas exogenous MT significantly upregulated these genes.

In starch degradation, the β-amylase gene *AgBAM* exhibited sustained high expression levels in both Salt and MTSalt groups, facilitating the conversion of starch into maltose to supply energy for metabolic activities under salt stress conditions. Compared to the Salt group, the MTSalt group showed upregulation of 4-α-glucanotransferase (*AgAG*), glucan endo-1,3-β-glucosidase (*AgEGLC*), and β-glucosidase (*AgBGLU*) genes, promoting glucose synthesis and thereby increasing the content of osmotic regulatory substances in celery. Furthermore, exogenous MT application enhanced the expression of hexokinase (*AgHK*) gene, regulating glucose phosphorylation to achieve energy supply. The endoglucanase genes *AgENU* (*Ag9G00724*, *Ag8G00587*) exhibited low expression in the Salt group but were upregulated in the MTSalt group. This suggests that MT may maintain the structural stability of celery cells under salt stress by regulating cellulose metabolism, thereby enhancing stress resistance.

### 2.8. qRT-PCR Validation of DEGs

**Figure 8:**
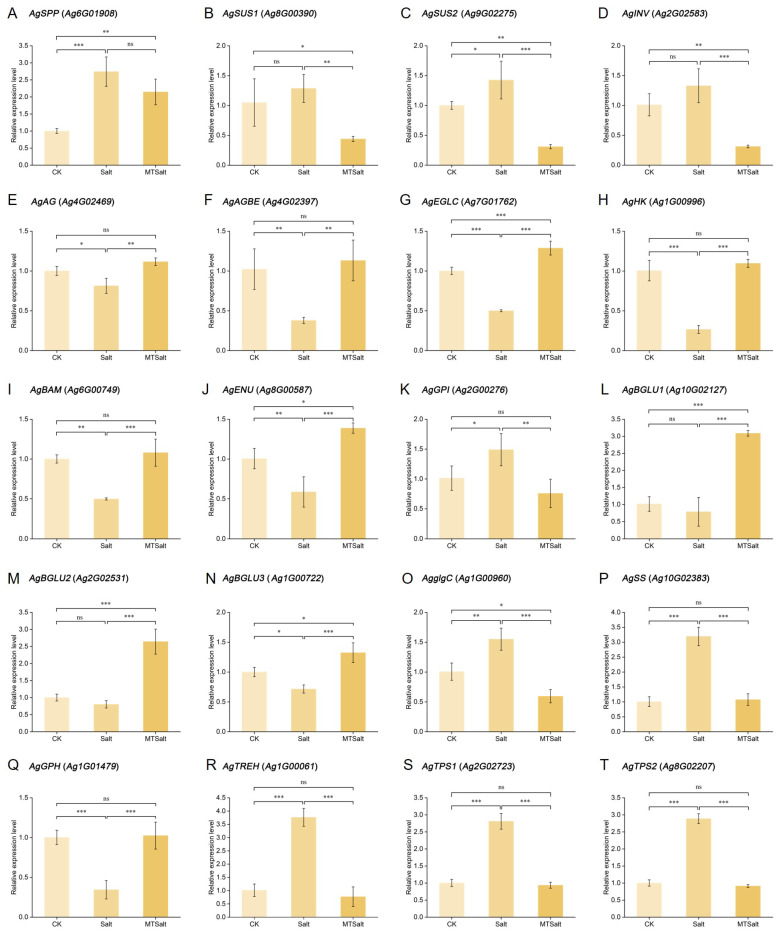
qRT-PCR validation of differentially expressed genes. (**A**–**T**) Relative expression level of genes in the starch and sucrose metabolism pathways. ns indicates no significant difference (*p* > 0.05), * indicates significant difference (*p* < 0.05), ** indicates highly significant difference (*p* < 0.01), and *** indicates extremely significant difference (*p* < 0.001).

To validate the reliability of RNA-seq data, qRT-PCR analysis was performed on 20 key genes selected from the starch and sucrose metabolism pathways. As shown in Figure 8, among the sucrose metabolism-related genes, *AgSPP* (*Ag6G01908*) and *AgSUS2* (*Ag9G02275*) exhibited significant upregulation under salt stress (Figure 8A,C). Conversely, exogenous MT treatment led to the downregulation of *AgSUS1* (*Ag8G00390*), *AgSUS2* (*Ag2G02279*), and *AgINV* (*Ag2G02583*) (Figure 8B–D). Among glucose and starch metabolism-related genes, salt stress resulted in the downregulation of *AgAG* (*Ag4G02469*), *AgAGBE* (*Ag9G02397*), and *AgEGLC* (*Ag7G01762*), while MT treatment reversed this suppression (Figure 8E–G). In the context of energy metabolism-related genes, *AgHK* (*Ag1G00996*) and *AgBAM* (*Ag6G00749*) maintained high expression levels in both the CK and MTSalt groups (Figure 8H,I). This suggests that MT may optimize energy supply in celery to respond to salt stress. Among cellulose metabolism-related genes, *AgENU* (*Ag8G00587*) was suppressed under salt stress but recovered after MT treatment (Figure 8J). The qRT-PCR results highly correlate with RNA-seq data, further corroborating the hypothesis that exogenous MT enhances stress response mechanisms.

## 3. Discussion

In this study, salt stress was found to significantly inhibit both the height and root length of celery seedlings, leading to symptoms such as leaf wilting and curling. This observation is consistent with the inhibitory effects of salt stress reported in other species such as chili peppers [27] and wheat [28]. Exogenous MT application effectively alleviated salt stress-induced growth inhibition in celery, consistent with studies in dragon fruit [29] and corn [30], where exogenous MT mitigated salt stress effects. Typically, salt stress induces a rapid accumulation of ROS within plant cells, resulting in oxidative stress. In this study, under salt stress, celery exhibited significantly increased MDA content and antioxidant enzymes (SOD, POD, CAT) activity, which promoted a marked increase in soluble protein and free proline content. Exogenous MT treatment further enhanced the activity of antioxidant enzymes and the accumulation of proline and soluble protein, while reducing MDA content. This indicates that MT alleviates oxidative damage by strengthening the antioxidant defense system, an effect that has also been reported in species such as apple [17], wheat [28], and cabbage [31]. In summary, exogenous MT may enhance the salt tolerance of celery through a synergistic mechanism that alleviates growth inhibition, boosts antioxidant capacity, and promotes osmotic regulation.

Salt stress inhibits the plant photosynthetic system at multiple levels, including decreased activity of photosynthesis-related enzymes, suppressed chlorophyll biosynthesis, and disrupted photosynthetic mechanisms [32,33,34,35]. In this study, salt stress caused significant decreases in chlorophyll a, chlorophyll b, and total chlorophyll content in celery seedlings, consistent with findings in potatoes [36] and peppers [27]. This suggests that salt stress may influence the activities of key enzymes involved in chlorophyll synthesis or accelerate chlorophyll degradation. Simultaneously, under salt stress, celery exhibited significantly lower values of ΦPSII, Fv/Fm, qL, and ETR compared to the CK group, while NPQ was markedly elevated—a finding consistent with the study on potato [36]. Exogenous MT treatment significantly restored chlorophyll content and PSII photochemical efficiency while mitigating the excessive increase in NPQ, indicating that MT plays a role in maintaining the integrity of the photosynthetic organelles. Studies on tomato have demonstrated that MT improves photosynthetic performance under salt stress by maintaining stomatal regulation, protecting chloroplast structure, stabilizing photosynthetic pigments, and enhancing electron transport chain activity [37,38]. Thus, MT protects the photosynthetic system through multiple pathways, thereby enhancing the photosynthetic capacity and carbon assimilation efficiency of celery under salt stress conditions.

GO enrichment analysis revealed that under salt stress, DEGs were primarily enriched in molecular function categories related to anhydrase activity. Following MT treatment, significant enrichment occurred in biological process categories such as carbohydrate metabolism and small-molecule metabolism pathways. KEGG pathway analysis revealed that DEGs were significantly enriched in pathways including photosynthesis, carbon metabolism, starch and sucrose metabolism, and plant hormone signaling. These pathways synergistically regulate carbon flux allocation, soluble sugar accumulation, and energy supply, providing the material and energy foundation for osmotic adaptation and physiological homeostasis under salt stress. Research indicates that MT regulates the expression of plant stress-related genes, thereby influencing their response to abiotic stress [12]. For example, MT upregulates the content of antioxidant enzymes and the expression of antioxidant-related genes [39]. Transcription factors play a crucial role in regulating plant responses to abiotic stress [40]. This study identified multiple differentially expressed transcription factor families including bHLH, ERF, NAC, WRKY, MYB, and HSF. Among these, genes belonging to the bHLH, ERF, and NAC families exhibited specific expression patterns after MT treatment, suggesting they may mediate the MT-induced response to salt stress. Similarly, in maize, MT was found to upregulate transcription factor genes such as *bHLH*, *MYB*, *APL*, and *HSF*, thereby regulating hormone signaling and enhancing salt tolerance [41]. Additionally, various transcription factors involved in MT-enhanced salt tolerance were identified in jujube [42]. Collectively, these results indicate that MT may constitute a gene expression regulatory network in celery’s response to salt stress by modulating a series of transcription factors.

Starch and sucrose metabolism plays a crucial role in osmotic regulation during plant stress responses [43]. In this study, under salt stress conditions, exogenous MT promoted the expression of *AgBAM*, accelerating starch degradation and thereby increasing the content of osmotic regulatory substances. In maize studies, exogenous MT increased sugar content by upregulating the amylolytic enzyme genes *ZmAMY* and *ZmBAM*, enhancing salt tolerance [20]. Under conditions of salt stress, plants under salt stress regulate the starch degradation pathway to promote sugar accumulation, thereby improving stress resistance [44,45,46]. Glucose, sucrose, and trehalose are crucial osmoregulatory substances. Salt stress promotes the expression of celery sucrose-degrading enzyme genes (*AgINV* and *AgSUS*) and trehalose synthesis, enhancing salt tolerance. In this study, salt stress promoted the expression of celery sucrose-degrading enzyme genes (*AgINV* and *AgSUS*) and trehalose synthase genes (*AgTPS*), while exogenous MT reversed this effect, inhibiting sucrose degradation and trehalose synthesis. Under salt stress, exogenous MT upregulated the expression of glucose synthase genes (*AgAG*, *AgEGLC*, and *AgBGLU*), promoting glucose synthesis. In maize studies, exogenous MT promoted sucrose degradation and inhibited trehalose synthesis in maize seeds, differing from the sucrose metabolism regulation observed in this study. This discrepancy may stem from species specificity or differing treatment conditions [20]. Research on watermelon indicates that sucrose accumulation contributes to enhanced salt tolerance [47]. Collectively, exogenous MT may regulate celery starch catabolism and sucrose and glucose accumulation to maintain cellular osmotic potential and enhance salt resistance.

## 4. Materials and Methods

### 4.1. Plant Material and Treatment

The celery cultivar “Ventura Xiqin” was utilized as the plant material in this study. Celery seeds were first soaked in warm water at 50 °C for 30 min, followed by immersion in cold water for 24 h. Subsequently, the seeds were placed in a seed incubator for germination. Approximately 7 days later, when 30–50% of the seeds had germinated, the seedlings were transplanted into 50-cell trays and placed back in the incubator. When the seedlings grew to 2-3 true leaves, the healthy seedlings with the same growth vigor were transplanted into 14 cm × 16 cm nutrient pots with a substrate mixture of nutrient soil, perlite, and vermiculite (3:1:1, *v*/*v*/*v*). The environmental conditions were maintained at 23 °C/18 °C (day/night), with a humidity level of 70%, a photoperiod of 14 h/10 h (day/night), and a light intensity of 1000 μmol∙m^−2^∙s^−1^ for routine management. Upon reaching a height of 20 cm, the celery seedlings with the same or similar growth vigor were moved into a hydroponic vessel with Hoagland’s solution for further growth. After a 7-day acclimation period, MT treatment was initiated. MT was first dissolved in a small amount of ethanol, resulting in a final ethanol concentration of less than 0.1% (*v*/*v*) and then diluted with distilled water to the target concentrations (0 μM, 100 μM, 200 μM, and 300 μM). Each treatment consisted of 20 seedlings. For 7 consecutive days, the solutions were sprayed evenly onto both adaxial and abaxial leaf surfaces of the seedlings until runoff occurred, with an application volume of approximately 10 mL per plant. All spraying was conducted in a wind-free environment to minimize solution drift. Control plant seedlings were sprayed with an equal volume of distilled water containing the same concentration of ethanol. On the 8th day, salt stress was induced by applying a 300 mM NaCl solution through irrigation, which was maintained for 10 days. At the end of the stress period, samples were collected for phenotypic observation, physiological assays, transcriptome sequencing, and expression analysis.

### 4.2. Measurement of Plant Growth and Physiological Indicators

Each physiological parameter was measured with three independent biological and technical repetitions. Plant height and root length were measured using a ruler and vernier caliper, respectively. For chlorophyll extraction, 0.3 g of fresh leaves was mixed with 95% ethanol solution and extracted in the dark for 24 h. After extraction, the absorbance was then measured at wavelengths of 649 nm and 665 nm using a spectrophotometer, and the contents of chlorophyll a, chlorophyll b, and total chlorophyll content were calculated according to the relevant formula [48]. For proline content, 0.2 g of fresh leaves was ground and centrifuged. The supernatant was subjected to an indophenol (IP) color reaction, followed by toluene extraction. Absorbance was measured at 520 nm, and proline content was quantified using a standard curve method [49]. For malondialdehyde (MDA), 1.0 g of fresh leaves was homogenized and centrifuged. The supernatant was used for the thiobarbituric acid (TBA) reaction, and the absorbance was measured at 532 nm to calculate MDA content [50]. Superoxide dismutase (SOD) activity was determined using the nitroblue tetrazolium (NBT) photoreduction inhibition method, measuring absorbance at 560 nm [51]. Catalase activity (CAT) was calculated by measuring H_2_O_2_ reduction at 240 nm [52]. Peroxidase (POD) activity was determined using the guaiacol method, measuring absorbance at 470 nm [53]. Soluble protein content was calculated using the Coomassie Brilliant Blue G-250 staining method, measuring absorbance at 595 nm and referencing a standard curve. Photosynthetic parameters (ΦPSII, Fv/Fm, NPQ, qL, and ETR) were measured using the LI-6400XT (LI-COR, Beijing, China) portable photosynthesis measurement system equipped with a 6400-40 leaf chamber fluorometer. Calibration was strictly performed according to the manufacturer’s instructions prior to measurement. Leaves were dark-adapted for 30 min in the integrated leaf holder. Measurements were conducted under photosynthetically active radiation (PAR) of 800 μmol·m^−2^·s^−1^, an atmospheric CO_2_ concentration of 400 μmol·mol^−1^, and a constant leaf temperature of 25 °C.

### 4.3. RNA Extraction, Library Construction, and Transcriptome Sequencing

Total RNA was extracted from celery fresh leaves from the control group (CK) and treatment groups (T_0_, T_2_) using Trizol reagent (Sangon Biotech, Shanghai, China). Each treatment group comprised three independent biological replicates. The RNA quality and quantity were evaluated using a Qubit2.0 Fluorimeter (Life Technologies, Carlsbad, CA, USA) and an Agilent Bioanalyzer 2100 System (Agilent Technologies, Santa Clara, CA, USA). High-quality total RNA was subsequently used as templates for reverse transcription into cDNA (~200 bp). The purified fragments were PCR-amplified and purified to construct the cDNA library. Paired-end sequencing was conducted on the Illumina HiSeq 2000 platform (Illumina, San Diego, CA, USA), with sequencing services provided by Novogene Co., Ltd. (Beijing, China). The transcriptome data were submitted to the China National Center for Bioinformation (CNCB) under the accession number PRJCA054076.

### 4.4. Functional Annotation of Differentially Expressed Genes (DEGs)

The celery reference genome sequence and corresponding gene annotation files were downloaded from the published celery genome database (http://tvir.bio2db.com/download.html) (accessed on 22 March 2025). Raw transcriptomic sequencing data underwent quality control preprocessing using Trimmomatic (v0.39) to remove reads containing adapter sequences, excessively high proportions of unknown bases, and low-quality bases. This yielded high-quality, clean reads for subsequent analysis (see Table S4 for details). Sequencing reads were aligned to the reference genome using HISAT2 (v2.2.1), and the raw read count matrix was generated with featureCounts (v2.0.6). Based on this matrix, differential expression analysis was performed using DESeq2 (v1.42.0) software. Significantly differentially expressed genes (DEGs) were identified using the criteria |log2(FoldChange)| ≥ 1 and *p*adj < 0.05. After transcript quantification normalization using FPKM (fragments per million reads per thousand bases), hierarchical clustering and K-means clustering analyses were performed on the identified significantly differentially expressed genes. Gene Ontology (GO) enrichment analysis (https://www.geneontology.org/, accessed on 10 May 2025) and Kyoto Encyclopedia of Genes and Genomes (KEGG) enrichment analysis (https://www.genome.jp/kegg/, accessed on 12 May 2025) were performed on the differentially expressed genes using clusterProfiler (v4.8.1) software. The transcription factor families of differentially expressed genes were identified and classified using the Plant Transcription Factor Database (PlantTFDB) (http://planttfdb.cbi.pku.edu.cn, accessed on 18 May 2025).

### 4.5. qRT-PCR Validation

The expression of selected DEGs was validated by qRT-PCR. Specific primers were designed using Primer Premier 6.0 (Table S5). Total RNA was extracted using Trizol reagent (Sangon Biotech, Shanghai, China), and cDNA was obtained using a reverse transcription kit (Beijing Qingke Biotechnology Co., Ltd., Beijing, China). qRT-PCR was performed using the BioEasy Master Mix (SYBR Green) kit (Hangzhou BioEasy Technology Co., Ltd., Hangzhou, China) and the C1000 Touch thermal cycler system (Bio-Rad, Hercules, CA, USA). *AgGAPDH* served as the reference gene, and the relative gene expression levels were calculated using the 2^−∆∆Ct^ method [54]. Each gene was analyzed in three biological replicates and three technical replicates.

### 4.6. Data Analysis and Processing

All measurements included three independent biological replicates, and data are presented as mean ± standard deviation. Statistical analysis was performed using SPSS 27 software. Significant differences among the multiple treatment groups were assessed by one-way analysis of variance (ANOVA). When the ANOVA indicated a significant effect (*p* < 0.05), Duncan’s multiple range test was applied for post hoc comparisons. Graphs were generated using Origin 2020.

## 5. Conclusions

This study systematically elucidates the complex mechanisms through which exogenous MT mitigates salt stress in celery. The physiological findings demonstrate that MT effectively counteracts salt-induced growth inhibition, oxidative damage, and photosynthetic decline by promoting the accumulation of osmotic regulators, such as proline and soluble proteins, enhancing the activity of antioxidant enzymes (SOD, POD, CAT) and safeguarding chlorophyll synthesis and photosystem II functionality. Gene expression analysis reveals that MT significantly modulates key genes involved in starch and sucrose metabolism pathways, thereby enhancing soluble sugar accumulation and osmotic regulation capacity. These findings provide novel insights into the role of MT in plant responses to salt stress.

## Data Availability

The original contributions presented in this study are included in the article/Supplementary Materials. Further inquiries can be directed to the corresponding authors.

## Supplementary Materials

The following supporting information can be downloaded at https://www.mdpi.com/article/10.3390/ijms27031299/s1.
