# Supplementary material for: Exogenous Melatonin Enhances the Salt Tolerance of Celery (Apium graveolens L.) by Regulating Osmotic Adaptation and Energy Metabolism via Starch and Sucrose Metabolic Pathways
Source: Int J Mol Sci. 2026 Jan 28;27(3):1299. doi: 10.3390/ijms27031299 (PMC12897849; doi:10.3390/ijms27031299)
Supplement: Supplementary file 1 [file ijms-27-01299-s001.zip › ijms-4082132-supplementary/Figure S1.pdf]

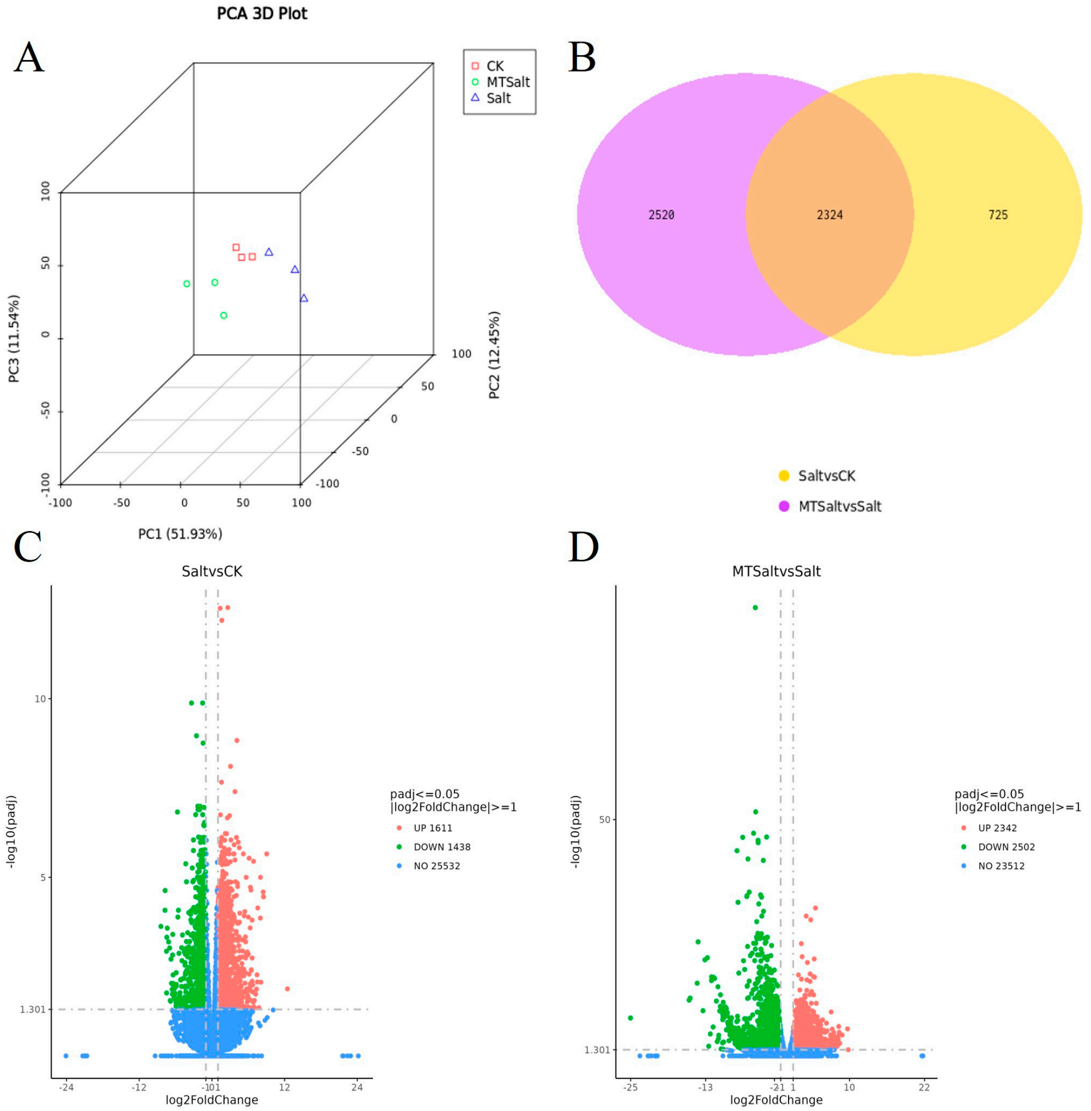


**Figure S1**. Overview of the gene expression profiles of different samples. (A) PCA score plots of transcriptome data for three groups (CK, Salt, MTSalt). (B) The Venn plot represents the number and proportion of DEGs that are commonly or uniquely expressed in pairwise comparisons. (C) DEGs volcano map in Salt vs CK group. (D) DEGs volcano map in MTSalt vs Salt group.
